# Aneurysm or diverticulum? You better look twice—Two rare faces of hypertrophic cardiomyopathy

**DOI:** 10.1007/s12471-023-01852-6

**Published:** 2024-02-22

**Authors:** Silvio Quick, Karim Ibrahim, Akram Youssef, Lorena Payo-Anez

**Affiliations:** Department of Cardiology, Angiology and Intensive Care, Klinikum Chemnitz gGmbH, Medical Campus Chemnitz of the Technische Universität Dresden, Dresden, Germany

**Fig. 1:**
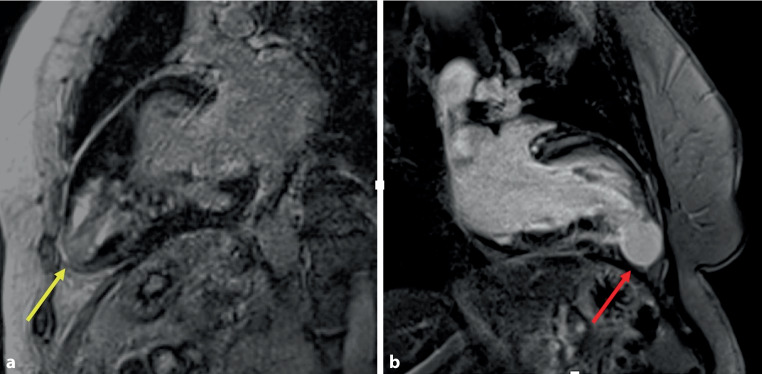
Two-chamber late gadolinium enhancement image of **a** patient with Yamaguchi syndrome and apical aneurysm showing fibrotic apical aneurysm (yellow arrow) and **b** patient with hypertrophic cardiomyopathy and congenital diverticulum without evidence of fibrosis (red arrow)

Hypertrophic cardiomyopathy (HCM) can manifest as rare pouch-like anomalies in the left ventricle, such as congenital diverticula or aneurysms, which have significant clinical implications. We present two distinct cases of this type of HCM. First, we saw a 67-year-old man with an HCM-related apical aneurysm, known as Yamaguchi syndrome (Fig. 1a, and see Videos 1 and 2 in Electronic Supplementary Material). Second, there was a 50-year-old woman who had a congenital left ventricular diverticulum, which was diagnosed by cardiac magnetic resonance imaging (MRI) (Fig. 1b, and see Videos 3 and 4 in Electronic Supplementary Material). Concerns about the potential rupture risks associated with diverticula prompted her to opt for surgical correction. Discriminating between diverticula and aneurysms hinges on both morphological characteristics and contractility patterns. Notably, congenital diverticula, characterised by their extremely thin walls, only exhibit nuanced contractile behaviours. Cardiac MRI plays a pivotal role, as it distinctly identifies the scar-like alterations inherent to aneurysms, a feature absent in diverticula. While traditional HCM approaches guide aneurysm treatment, diverticulum management is case-specific, highlighting the need for further clinical research.

### Supplementary Information

**Video 1** Two-chamber view cine sequence of patient with Yamaguchi syndrome showing midventricular obstruction and apical aneurysm

**Video 2** Four-chamber view cine sequence of patient with Yamaguchi syndrome showing midventricular obstruction and apical aneurysm

**Video 3** Two-chamber view cine sequence of patient with hypertrophic cardiomyopathy and congenital diverticulum. Diverticular contractility is less evident as a visible inward motion but rather as a subtle early systolic increase in thickness

**Video 4** Four-chamber view cine sequence of patient with hypertrophic cardiomyopathy and congenital diverticulum. Diverticular contractility is less evident as a visible inward motion but rather as a subtle early systolic increase in thickness

